# Autocrine production of reproductive axis neuropeptides affects proliferation of canine osteosarcoma in vitro

**DOI:** 10.1186/s12885-019-5363-4

**Published:** 2019-02-18

**Authors:** Marcus A. Weinman, Jacob A. Fischer, Dakota C. Jacobs, Cheri P. Goodall, Shay Bracha, Patrick E. Chappell

**Affiliations:** 1 Department of Clinical Sciences, Carlson College of Veterinary Medicine, Oregon State University, Corvallis, OR 97331 USA; 2 Department of Biomedical Sciences, Carlson College of Veterinary Medicine, Oregon State University, Corvallis, OR 97331 USA

**Keywords:** Osteosarcoma, GnRH, Kisspeptin, Autocrine, Proliferation

## Abstract

**Background:**

Osteosarcoma strikes hundreds of people each year, of both advanced and younger ages, and is often terminal. Like many tumor types, these bone tumors will frequently undergo a neuroendocrine transition, utilizing autocrine and/or paracrine hormones as growth factors and/or promoters of angiogenesis to facilitate progression and metastasis. While many of these factors and their actions on tumor growth are characterized, some tumor-derived neuropeptides remain unexplored.

**Methods:**

Using validated canine osteosarcoma cell lines in vitro*,* as well as cells derived from spontaneous tumors in dogs, we explored the autocrine production of two neuropeptides typically found in the hypothalamus, and most closely associated with reproduction: gonadotropin-releasing hormone (GnRH) and kisspeptin (Kiss-1). We evaluated gene expression and protein secretion of these hormones using quantitative RT-PCR and a sensitive radioimmunoassay, and explored changes in cell proliferation determined by MTS cell viability assays.

**Results:**

Our current studies reveal that several canine osteosarcoma cell lines (COS, POS, HMPOS, D17, C4) synthesize and secrete GnRH and express the GnRH receptor, while COS and POS also express *kiss1* and its cognate receptor. We have further found that GnRH and kisspeptin, exogenously applied to these tumor cells, exert significant effects on both gene expression and proliferation. Of particular interest, kisspeptin exposure stimulated GnRH secretion from COS, similarly to the functional relationship observed within the neuroendocrine reproductive axis. Additionally, GnRH and kisspeptin treatment both increased COS proliferation, which additionally manifested in increased expression of the bone remodeling ligand *rankl* within these cells. These effects were blocked by treatment with a specific GnRH receptor inhibitor. Both neuropeptides were found to increase expression of the specific serotonin (5HT) receptor *htr2a*, the activation of which has previously been associated with cellular proliferation, suggesting that production of these factors by osteosarcoma cells may act to sensitize tumors to circulating 5HT of local and/or enteric origin.

**Conclusions:**

Here we report that kisspeptin and GnRH act as autocrine growth factors in canine osteosarcoma cells in vitro, modulating RANKL and serotonin receptor expression in a manner consistent with pro-proliferative effects. Pharmacological targeting of these hormones may represent new avenues of osteosarcoma treatment.

**Electronic supplementary material:**

The online version of this article (10.1186/s12885-019-5363-4) contains supplementary material, which is available to authorized users.

## Background

Osteosarcoma is the most common skeletal tumor in humans, striking a significant portion of people in their mid-20s, with a very low survival rate even with chemotherapeutic intervention [1, 2]. Incidences of canine osteosarcoma are even higher, and the disease shares many characteristics with the human equivalent [3]. Amputation and adjuvant chemotherapy are the standard of care in dogs; however poor outcomes often result due to pulmonary metastasis [4]. To better address this disease in both species and potentially design novel targeted therapies, an enhanced understanding of local and systemic factors contributing to disease progression is essential. Recent evidence suggests an important role for serotonin (5HT) in normal bone physiology, and possibly an alteration of the role of 5HT in progression of osteosarcoma. Osteoblasts, osteocytes and osteoclasts express functional 5HT receptors (5HTR), and several remodeling pathways exhibit a dependency on circulating 5HT [5–7]. In investigating the role of 5HT in bone cancer, our group previously demonstrated a role for 5HTR in canine osteosarcoma in vitro using validated canine osteosarcoma cell lines (COS) [8]. In these studies, COS cells exhibited dose-dependent increases in proliferation following 5HT treatment. It was further demonstrated that expression of the 2A isoform of 5HTR (5HTR2A) was substantially increased in malignant cells in comparison to normal canine osteoblasts, ostensibly increasing the sensitivity of tumor cells to circulating 5HT [8].

The majority of circulating 5HT originates in enterochromaffin cells, and while this might play a role in regulating tumor growth rate, local autocrine and paracrine factors within tumor cells and surrounding stroma also likely modulate tumor progression, both by decreasing dependency on circulating factors or by sensitizing tumor cells to these factors. We posited that, similarly to what has been observed in other progressing tumor subtypes [9–11], osteosarcoma tumors may develop a neuroendocrine phenotype, synthesizing peptides often found in the hypothalamus. Gonadotropin-releasing hormone (GnRH) synthesis and secretion has been documented mostly in reproductive cancers such as endometrial, ovarian, breast and prostatic cancers [12–14]. GnRH receptor (GnRHR) expression was also demonstrated in the aforementioned reproductive cancers, with highly variable signaling effects on cancer proliferation [12]. Under normophysiologic conditions within the reproductive axis, continuous GnRHR stimulation results in receptor desensitization and downregulation of expression, while pulsatile GnRH secretion has little effect on GnRHR sensitivity and *gnrhr* transcript levels [12]. In cancers, the expression of GnRH has been associated with poor prognosis, but expression and production of this neuropeptide and its receptor in osteosarcoma remains unexplored. Additionally, the neuropeptide kisspeptin, indispensable for pubertal progression and fertility, may be synthesized in tumor cells (or neighboring stromal cells) and modulate tumor cell function of osteosarcoma [15–17]. This factor was formerly known as metastin, and was originally characterized in multiple tumor subtypes. While it is clear that kisspeptin plays an important role in initiating secretion of GnRH in the brain, the role of kisspeptin and its cognate receptor Kiss1R (a.k.a. GPR54) and their role in malignancy in cancer is still under investigation.

The bone remodeling system is governed by the protein triad of RANK (Receptor activator of Nuclear Factor κ-B), RANKL (RANK-Ligand), and OPG (Osteoprotegerin). In normal remodeling, RANKL binds its cognate receptor, RANK, to stimulate osteoclastic maturation, activity, and subsequent bone resorption. Osteoblasts transiently synthesize RANKL and OPG, depending on their state of differentiation and exposure to afferent stimuli, while osteoclast precursors express RANK [18]. The OPG protein, also synthesized by osteocytes, functions as a decoy receptor for RANKL, such that the ratio of RANKL to OPG effectively dictates the amount of osteoclast formation and activity at any given time [19]. This homeostasis can be modulated by multiple circulating hormones. Estrogen, an ovarian steroid hormone, exerts a particularly profound effect on bone remodeling. Previous studies have shown that treatment of human osteoblasts with physiologic concentrations of estradiol increased estrogen receptor and OPG expression, which downregulates osteoclastic activity by preventing RANK-RANKL binding [20]. This response to estrogen creates a regulatory mechanism through which osteoblasts can modulate homeostasis of bone deposition and resorption dependent upon cycling steroid levels. This system becomes dysfunctional in tumor cells, both primary and secondary. In support of this, Good et al. performed a prospective study of cancer patients with primary and metastatic bone tumors that showed, via immunohistochemistry, that both types expressed RANKL [21]. Increases of the RANKL to OPG ratio by tumor cells, regardless of cellular origin, shifts the balance towards bone resorption and possible osteolysis.

Furthermore, RANKL-RANK binding activates the potent transcription factor NF-κB, which induces expression of an array of anti-apoptotic genes, promotes cell cycle progression, increases invasiveness, promotes angiogenesis, and induces inflammation [22]. Increases of the RANKL/OPG ratio therefore result in elevated NF-κB activation. While effects of estrogen have been explored, possible relationships between peptidergic reproductive hormones GnRH and kisspeptin and the RANK-RANKL-OPG system are unclear, since under normophysiologic conditions, GnRH is not found in the circulation. In a model of breast cancer, GnRH was shown to decrease in vitro *rankl* expression in RANKL^+^ breast cancer cells co-cultured with human osteoblasts [23]. Regarding known interactions between GnRH/kisspeptin and NF-κB, Zhang et al. previously showed that NF-κB activation represses *gnrh* expression within the murine hypothalamus [24]. In the context of osteosarcoma, however, it is currently unknown whether GnRH or kisspeptin alter the function of the RANK-RANKL-OPG system, change NF-κB activation, or experience regulation of expression and secretion via NF-κB.

In the current study, we sought to examine 1) if these typically reproductive neuropeptides (kisspeptin and GnRH) are synthesized in canine osteosarcoma cell lines, 2) if GnRH may be secreted by these tumor cells, and 3) what effects autocrine production of these peptides could have on tumor growth and bone remodeling physiology. Additionally, we explored if the stimulatory effects of kisspeptin on GnRH synthesis and secretion found normally in the neuroendocrine reproductive axis are recapitulated in the local tumor microenvironment, including whether this relationship could be modulated by estrogen. Lastly, we investigated if these neuropeptides could modulate the previously characterized effects of 5HT on osteosarcoma growth rate.

## Methods

### Validation of cell lines

COS, HMPOS, POS, D17, C4 are well-characterized immortal canine osteosarcoma cell lines. COS were provided to SB by Dr. Vilma Yuzbazian (Michigan State Univ.). HMPOS (RRID:CVCL_L355), POS (RRID:CVCL_L413), D17 (RRID:CVCL_1916), and SAOS (RRID:CVCL_0548) cells were provided by ATCC. C4 (clone 4) canine osteosarcoma line was derived by Dr. Bernard Seguin at UC Davis, and provided to the Oregon State University Carlson College of Veterinary Medicine in 2014. Canine osteogenic progenitor cells (OPCs), which are committed (but not differentiated) mesenchymal stem cells, were purchased from Cell Application Inc., San Diego, CA. GT1–7 mouse hypothalamic neuronal line was provided by Dr. Pamela Mellon (UCSD). Validation markers for COS, POS, and HMPOS include western blot confirmation of bone-specific alkaline phosphatase, as well as osteoblast-specific markers *runx2* and *sp7*, confirmed by RT-PCR. COS, POS, HMPOS, and SAOS cells were mycoplasma tested and confirmed clean as of April 2017.

### Cell culture and treatment

Osteosarcoma tumor cells were grown in RPMI-1640 media supplemented with 10% fetal bovine serum (FBS), L-glutamine, sodium pyruvate, HEPES buffer, 100 μg/mL Streptomycin, 100 units/mL Penicillin, and 50 μg/mL Gentamicin. Osteogenic progenitor cells (OPCs) were grown in a proprietary media supplied by the manufacturer (Cell Application Inc., San Diego, CA). GT1–7 cells (immortalized mouse GnRH neurons) were grown in DMEM with 10% FBS and 100 μg/mL Streptomycin and 100 units/mL Penicillin. All cells were incubated in 37 °C at 5% CO_2_ and grown to confluence for experiments.

### PCR and sequencing

Genomic DNA and total RNA were extracted from plates of confluent cells using the QIA DNA Mini-Kit (Qiagen) and TriZol, respectively. Specific primers were designed to amplify within the coding regions of *β-actin, gpr54, gnrh, htr2a, kiss1, gnrhr, rank, rankl, opg,* and *ywhaz*. PCR products were validated via gel electrophoresis (2% agarose) with ethidium bromide. Sequencing was performed by the Center for Genome Research and Biocomputing at Oregon State University; sequences were confirmed against the published sequences at the National Center of Biotechnology Information website.

### Real-time RT-PCR

One microgram total RNA extracted from OPC, GT1–7, COS, HMPOS and POS, via standard methods, was converted to cDNA using High Capacity cDNA Reverse Transcription Kit (Life Technologies, Eugene, OR). Real-time PCR compatible primer pairs were designed for canine *β-actin, kiss1*, *gpr54*, *gnrh*, *gnrhr*, *htr2a, rank, rankl, opg, and ywhaz,* (Additional file 2**:** Table S1) using the NCBI’s Primer-BLAST (http://www.ncbi.nlm.nih.gov/tools/primer-blast/). PCR was executed with Power SYBR Green (Life Technologies, Eugene, OR) using a StepOnePlus real-time thermal cycler (ABI/ThermoFisher). Relative gene expression was calculated by the 2^−ΔΔCT^ method in relation to the endogenous expression of *ywhaz* and/or *β-actin* at each time point. Values shown are normalized to vehicle (0.1–0.01% DMSO) control.

### Cell viability assay

Viability assays were performed with CellTiter 96® Aqueous One Solution (Promega, Madison, WI), which is a MTS tetrazolium assay. Cells were plated in triplicate on 96-well culture plates at 1 × 10^4^ cells per well and incubated in serum free media for 24 h, after which time the media was replaced by a fresh light-treated media that contained 0.1% DMSO for the control untreated groups or 6.0 μM 5HT, following 24 h of 1.0 nM GnRH pre-treatment. Treatments not involving 5HT were carried out with as described with DMSO for the untreated control groups or 1.0 nM GnRH, 10.0 nM Kisspeptin, 10.0 nM Teverelix, 100.0 pM 17β-estradiol, or listed combinations of the above for treatment groups. Cells were processed following manufacturer’s instructions and absorbance was measured at 490 nm on a microplate reader.

### Statistical analyses

Cell viability data from the proliferation assay were analyzed using a generalized linear model to account for variation due to time and concentration of 5HT and GnRH. The resulting model was then subjected to one-way ANOVA analysis, followed by Tukey’s Honest Significant Difference test to compare the effects of dose increases separately or in combination at differing times. ANOVA and Tukey’s HSD analyses were carried out using GraphPad Prism software. Statistical significance was assigned for calculated values of *p* < 0.05.

### Cell perifusion

COS were transferred to adherent Cytodex 3 beads and incubated in petri dishes for 24–96 h. Cells on beads were loaded into 1.0 cc columns in a 37.0 °C incubator and perifused with serum-free RPMI at a flow rate of 100 μL/min using a Gilson peristaltic pump. One milliliter fractions were collected every 10 min using refrigerated Gilson fraction collectors. Perifusates were frozen at − 20 °C until GnRH radioimmunoassay. Media containing either 10.0 nM kisspeptin-10, 6.0 μM serotonin, or 12.5 μM ritanserin was perifused over COS cells for two hour intervals, followed by washout for 2–4 h.

### Radioimmunoassay

Media from cells collected in static incubation were concentrated 1:5 and resuspended in 100 μL PBS-G/replicate. One hundred microliters media from perifusate were assayed directly using the EL-14 primary antibody (gift from M. Kelly and O. Ronnekleiv). Intra- and interassay CV was 4.9 and 6.7%, respectively.

### Immunoblotting

Total protein concentrations from COS, OPC, and GT1–7 cell lysates were determined via BCA assay kit (Pierce Biotechnologies, Rockford, IL). Plates were read using a SkanIt plate-reader (Thermo Fisher Scientific, Waltham, MA) at absorbance of 562 nm. Twenty-five micrograms of protein were separated on a 10% polyacrylamide gel and transferred to a nitrocellulose membrane. Primary antibodies for GPR54/Kiss1R (Alamone, Jerusalem, Israel) were used to probe blots. Bound primary antibody was probed by goat anti-rabbit HRP and detected with Supersignal West Pico Chemiluminescent Substrate (Thermo Scientific, Waltham, MA). The blots were scanned on an ImageQuant LAS4000 (GE Healthcare Life Sciences, Marlborough, MS). Anti-alpha-tubulin followed by goat anti-rabbit HRP (Santa Cruz Biotechnology, Dallas, TX) were used to assess protein loading and normalize band intensities.

## Results

### Expression of reproductive neuropeptides and bone remodeling factors in canine osteosarcoma cell lines

**Fig. 1:**
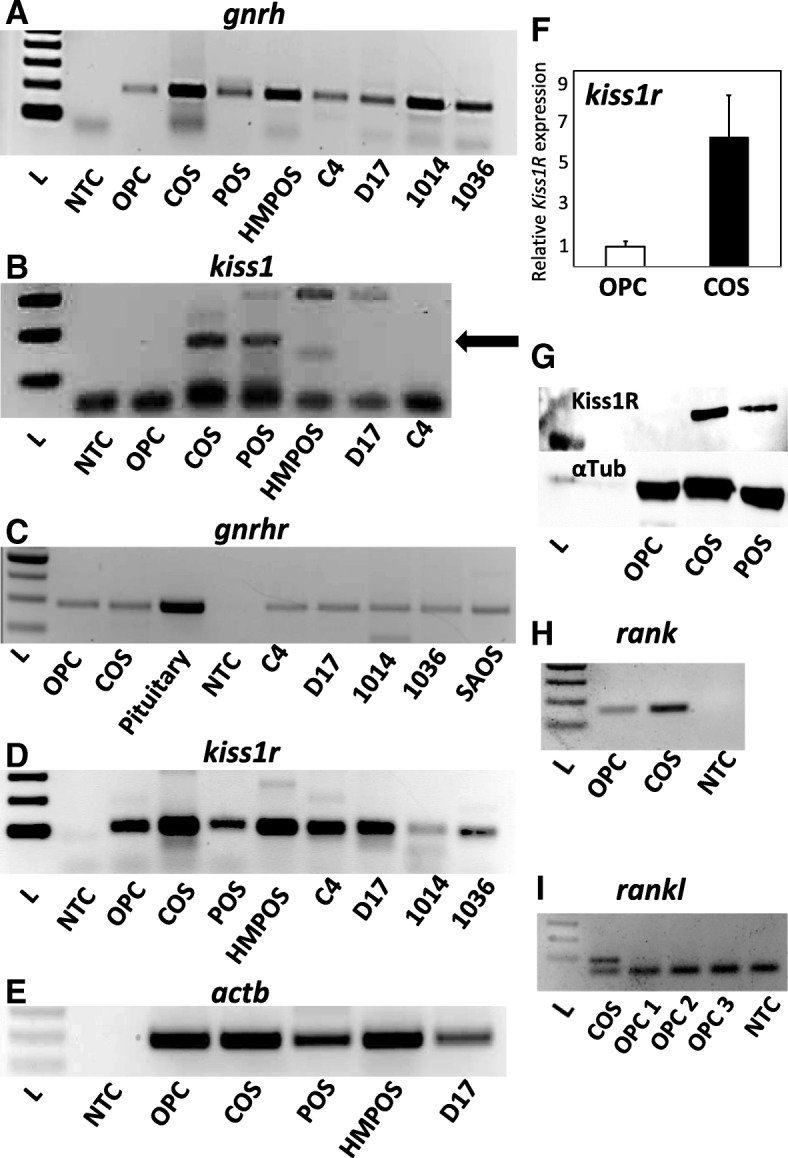
**(a)** *gnrh* **(b)** *kiss1,* **(c)** *gnrhr*, and (**d**) *gpr54/kiss1r* mRNA expression in normal canine osteoblasts (OPC), multiple osteosarcoma cell lines (COS, POS, HMPOS, C4, D17, human SAOS) and clinical patient-derived samples (1014, 1036). Arrow in (**b**) indicates *kiss1* band. PCR primers used can be found in Additional file 2 Table S1. **e** β-actin (*actb*) expression in OPC, COS, POS, HMPOS, and D17 cells. **f** Relative levels of *cKiss1R* expression in COS vs. OPC. **g** Immunoblot of Kiss1R protein (54kD, upper band) in normal canine osteoblasts (OPC) and osteosarcoma lines (COS, POS); α-tubulin (42kD, lower band) was also probed as a control. **h** RT-PCR expression of *rank* in COS vs. OPC. **i** RT-PCR expression of *rankl* in COS vs. OPC (3 preparations). NTC indicates RNA (no cDNA template) control

To explore if canine osteosarcoma cells may employ reproductive neurohormones and their receptors as autocrine/paracrine factors, we probed established tumor cell lines for expression of canine *gnrh* and *kiss1*, and their respective cognate receptors *gnrhr* and *gpr54/kiss1R*, using RT-PCR (Fig. 1a-d). *GnRH* expression was observed in all lines examined, found also in primary canine tumors acquired from the Oregon State University College of Veterinary Medicine BioBank, and at lower levels in normal canine osteogenic progenitor cells (OPC) (Fig. 1a, e). Low basal levels of *kiss1* expression were observed in COS and POS lines (Fig. 1b), but not in D17, HMPOS, or OPCs. Pituitary-typical *gnrhr* expression was also observed in canine osteosarcoma lines COS, C4, and D17, and primary tumor samples, in addition to OPC (Fig. 1c). *Kiss1r* (*gpr54*) expression was observed in all osteosarcoma cells tested, primary samples, and normal OPCs (Fig. 1d). Isolated amplicons from these samples were confirmed by Sanger sequencing, which showed near-complete sequence homology (data not shown). GnRH receptor was expressed at similar levels among cultured cell lines, suggesting a threshold presence of receptor to respond to exogenously-applied GnRH, which exerted multiple effects on gene expression and proliferation in COS, effects that were blocked by the GnRHR inhibitor, Teverelix (see below).

Noting potential quantitative differences in *kiss1r* between COS and OPC using RT-PCR, we quantified expression and compared levels between this tumor line and normal canine osteogenic progenitor cells. Real-time RT-PCR results reveal that expression of *kiss1r* is present at elevated levels (~ 7-fold higher) in COS compared to normal osteogenic progenitor cells (Fig. 1f), which accompanied by absent *kiss1* expression in the latter cells (Fig. 1b), suggests a potential aberration of the kisspeptin peptide/receptor system in this type of cancer (Fig. 1d, e). Immunoblotting confirmed the presence of translated Kiss1R in two canine osteosarcoma lines, COS and POS, while Kiss1R protein was not detected in osteogenic progenitor cells (Fig. 1g). RT-PCR was also used to determine *rank* and *rankl* expression in COS cells and OPCs. COS cells express both *rank* and *rankl*, while osteogenic progenitor cells were found to express *rank* but not *rankl* (Fig. 1h, i), potentially because *rankl* expression is typically induced by stromal signals that promote osteoblastic differentiation of osteogenic progenitor cells. Additionally, *rank* expression in normal osteoblasts has been observed previously [25], and this pattern of *rank*/*rankl* expression in our OPCs may reflect a particular state of incomplete differentiation.

Using primer sets listed in Additional file 2**:** Table S1, we were able to sequence the complete coding region of canine *kiss1.* The sequence is 100% homologous to that which had been recently submitted to GenBank (accession number KJ512885) by Reynaud, et al. Nucleotide and amino acid sequence comparison of *kiss1* and kisspeptin are found in Additional file 1**:** Figure S1, demonstrating that canine kisspeptin is unique. A previous study [26] also found that the predicted protein and mRNA sequence for kisspeptin in the dog was substantially different from kisspeptin in other species. Using real-time qPCR, we noted *kiss1* expression in a two osteosarcoma cell lines (COS and POS), but not in normal osteogenic progenitor cells (OPC).

### GnRH is secreted from COS, and this secretion is stimulated by kisspeptin

**Fig. 2:**
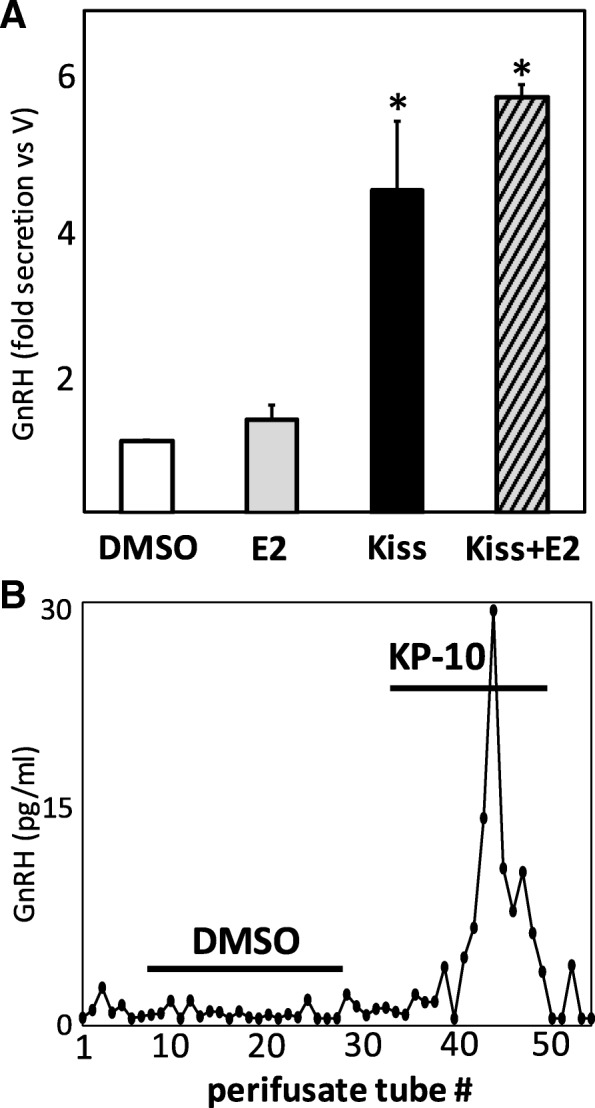
**a** GnRH secretion in static incubation from media of COS in vitro treated for 4 h with 17β-estradiol (100.0 pM), kisspeptin-10 (10.0 nM), or a combination of E2 and kisspeptin-10. **b** Representative plot of GnRH secretion from perifused COS treated with either 0.05% DMSO vehicle or 10.0 nM kisspeptin-10. Lines indicate duration of exposure to drug/vehicle

To determine if the mature GnRH decapeptide may also be synthesized and secreted by COS to potentially act as an autocrine factor, we measured GnRH secretion from these tumor cells in multiple culture formats using a sensitive radioimmunoassay [27]. Briefly, COS were grown both in static incubation in tissue culture flasks, as well as on adherent Cytodex 3 beads for subsequent cell perifusion and fraction collection [27]. As observed in Fig. 2, COS secrete low (~ 2–5 pg/ml) but detectible concentrations of GnRH into the media under basal conditions. Strikingly, this secretion from COS is stimulated 4–5 fold by 10.0 nM human kisspeptin-10 treatment (18.2 ± 2.8 pg/ml), both in the presence and absence of 100.0 pM 17β-estradiol (22.5 ± 0.8 pg/ml; *, *p* < 0.05; *n* = 6, Fig. 2a). Secretion of GnRH was also noted from COS grown in cell perifusion format, appearing pulsatile, with similar secretory parameters observed in immortalized GnRH-secreting GT1–7 [27, 28]. In this format as well, 10.0 nM kisspeptin-10 was found to rapidly and transiently stimulate GnRH secretion from perifused COS in comparison to DMSO vehicle treatment (KP-10: 28.5 ± 1.9 pg/ml vs. DMSO: 3.7 ± 0.4 pg/ml; *, *P* < 0.05, *n* = 6), which had no observable effect on GnRH secretory baseline (representative perifusion, Fig. 2b).

### Treatment of COS with exogenous GnRH and kisspeptin alters cellular viability

**Fig. 3:**
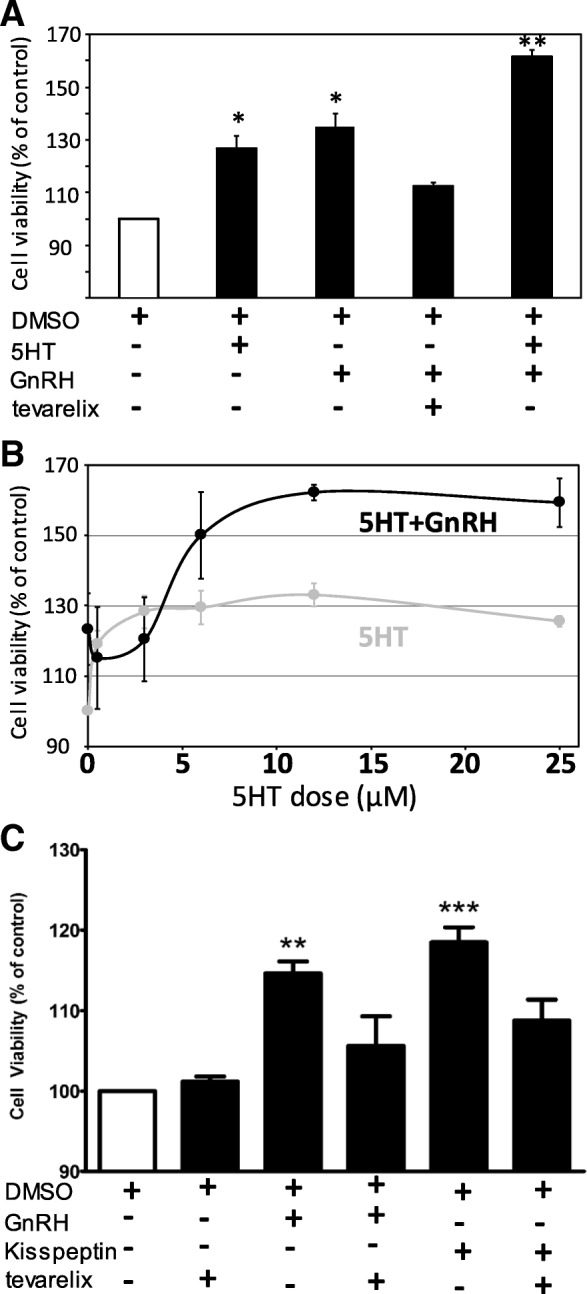
**a** Cell viability of COS following 24–48 h of treatment with 12.5 μM 5HT (2nd bar, *, *p* < 0.05; *n* = 6), 1.0 nM GnRH (3rd bar, *, *p* < 0.05; *n* = 6), 1.0 nM GnRH with 10.0 nM Teverelix (GnRHR inhibitor; 4th bar), or GnRH and 5HT co-treatment (last bar, **, *p* < 0.01; n = 6). **b** Cell viability of COS (as % of DMSO control) following increasing doses of either 5HT alone (grey line) or GnRH+5HT (black line). **c** Cell viability of COS following 24 h of treatment with 10 nM kisspeptin, 10 nM Teverelix, 10 nM each Kisspeptin+Teverelix, 1.0 nM GnRH, or 1.0 nM GnRH + 10 nM Teverelix (**, *p* < 0.01; *n* = 6; ***, *p* < 0.001; *n* = 6)

To investigate if exposure to GnRH or kisspeptin could modulate tumor growth in vitro, we performed cell viability assays following treatment of COS with these peptides. MTS assays revealed a dose-dependent effect of GnRH on COS proliferation, with lower doses of GnRH (0.1–1.0 nM) exerting significant (~ 35% increase ±6.4%; *p* < 0.05; *n* = 6) increases in COS proliferation above vehicle treatment (Fig. 3a). We previously demonstrated that exogenously applied serotonin (5HT) could alter proliferation of COS in vitro [8]. To determine if GnRH and/or kisspeptin may potentiate or counter these effects of 5HT on tumor growth, COS were treated with doses (0.5, 3, 6, 12, and 25 μM) of this indoleamine for 24 h following treatment with either 1.0 nM GnRH or 10.0 nM kisspeptin, and proliferation was again evaluated via MTS assay. Sequential co-incubation of COS with 5HT and 10.0 nM GnRH resulted in potentiation of COS proliferation above 5HT treatment alone, particularly at doses of 6–25 μM 5HT (*, *p* < 0.05; **, *p* < 0.01; n = 6/treatment; Fig. 3 **a, b**). Interestingly, KP-10 co-treatment with 5HT exerted no additional effects on cell growth above that observed for 5HT treatment alone (data not shown). To further examine how these neuropeptides may alter tumor growth, additional MTS assays were performed with GnRH, kisspeptin, and the GnRH receptor inhibitor Teverelix, both individually and in combination. These treatments illustrate that kisspeptin and GnRH both increase COS proliferation above control, while antagonism of GnRHR via co-incubation with Teverelix (10.0 nM) attenuated both GnRH- and Kisspeptin-stimulated increases in proliferation (Fig. 3c, **, p < 0.01; ***, *p* < 0.001; *n* = 5/treatment), suggesting that kisspeptin may increase COS cell proliferation via GnRH-dependent mechanisms.

### Hormone treatment modifies the bone remodeling system through potentiation of rankl expression

**Fig. 4:**
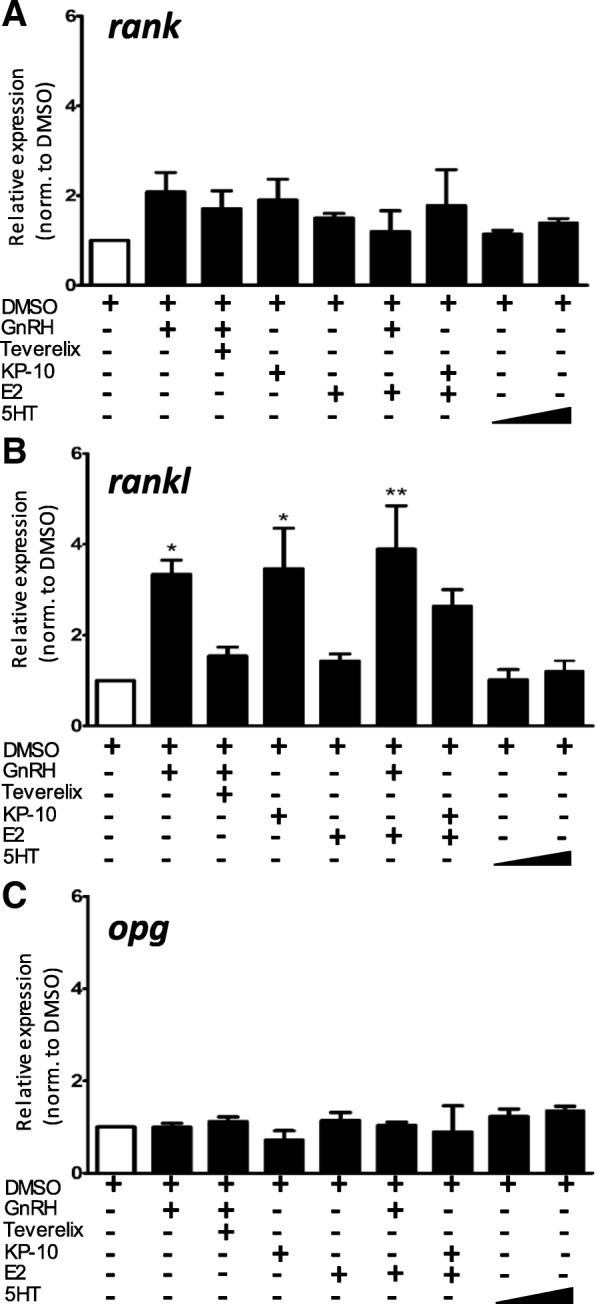
**a** Changes in *rank* gene expression after treatment with 1.0 nM GnRH, 1.0 nM GnRH + 10.0 nM Teverelix, 10.0 nM kisspeptin, 100.0 pM 17β-estradiol, 1.0 nM GnRH + 100 pM 17β-estradiol, 10.0 nM kisspeptin + 100.0 pM 17β-estradiol, or 5HT (3.0 μM and 12.0 μM). **b** Changes in *rankl* gene expression after treatment with hormones described above (*, *p* < 0.05; **, *p* < 0.01, *n* = 6/treatment). **c** Changes in *opg* gene expression after treatment with hormones described above

To further investigate mechanisms of how 5HT, GnRH, kisspeptin, and estrogen treatment may alter growth of COS cells, we performed cell culture treatments with these hormones and quantified changes in gene expression for *rank, rankl,* and *opg* at 4-h post-treatment. Both GnRH and kisspeptin increase *rankl* gene expression (Fig. 4b, *, *p* < 0.05, *n* = 6/treatment). The presence of Teverelix in combination with GnRH reduced *rankl* expression by 50% in comparison to GnRH alone. These results suggest that GnRH and kisspeptin favor an increase in the RANKL/OPG ratio, which would increase downstream NF-κB activation. Gene expression for *rank* trends upwards (*p* < 0.057) towards a 2-fold increase after treatment (Fig. 4a), suggesting that, over time, *rank* expression may continue to increase with GnRH and kisspeptin treatment. No significant changes in *opg* expression were observed (Fig. 4c). Not surprisingly, in these transformed tumor cells, estrogen fails to exert its normal physiologic effect of increasing *opg* expression to counteract increasing levels of RANKL protein. Serotonin treatment, at either low (3.0 μM) or high (12.0 μM) doses, had no effect on expression levels of any of the bone remodeling factors.

### GnRH and kisspeptin treatment increases expression of 5HT receptor htr2A

**Fig. 5:**
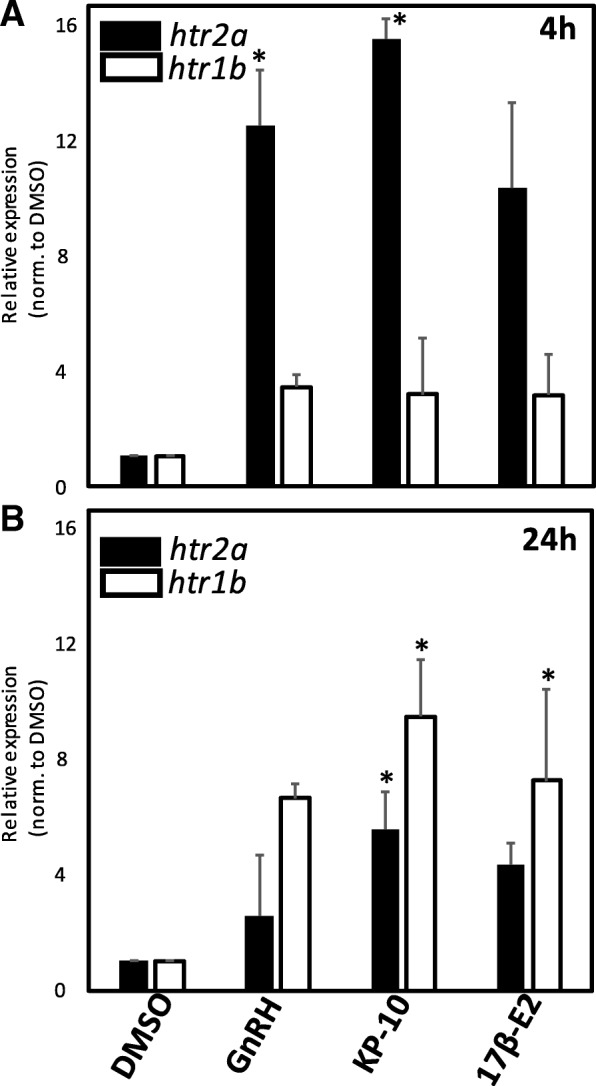
**a** Relative expression of *htr2a* (black bars) and *htr1b* (white bars) following 4 h treatment with 1.0 nM GnRH, 10.0 nM kisspeptin-10, or 100.0 pM 17β-E2. **b** Relative expression of *htr2a* and *htr1b* following 24 h (right panel) treatment with 1.0 nM GnRH, 10.0 nM kisspeptin-10, or 100.0 pM 17β-E2. (*, *p* < 0.05, *n* = 5 for each treatment group)

We recently demonstrated that exogenous 5HT treatment of COS, putatively representative of enteric 5HT in vivo, could modulate expression levels of the serotonin receptors *htr2A* and *htr1B*, leading to changes in proliferation via the constitutive phosphorylation of ERK and CREB [8]. To determine if exposure to these reproductive neuropeptides may potentiate the pro-proliferative effects of 5HT, we evaluated the effects of exogenous GnRH and kisspeptin-10 treatment on levels of the specific *htr2A* isoform of 5HT receptor. Real-time RT-qPCR (Fig. 5) revealed that 1.0 nM GnRH and 10.0 nM kisspeptin-10 increased *htr2a* expression 2- (at 24 h) to 8-fold (at 4 h) over levels observed in vehicle-treated COS (*, *p* < 0.05; *n* = 6), suggesting these peptides may potentiate 5HT-stimulated growth at least in part by increasing abundance of the pro-proliferative receptor isoform HTR2A. Kisspeptin treatment also increased expression of *htr1b*, with significant increases (*, p < 0.05; n = 6) not observed until 24 h following exposure, in contrast to the transiently robust increases observed for *htr2a* expression.

**Fig. 6:**
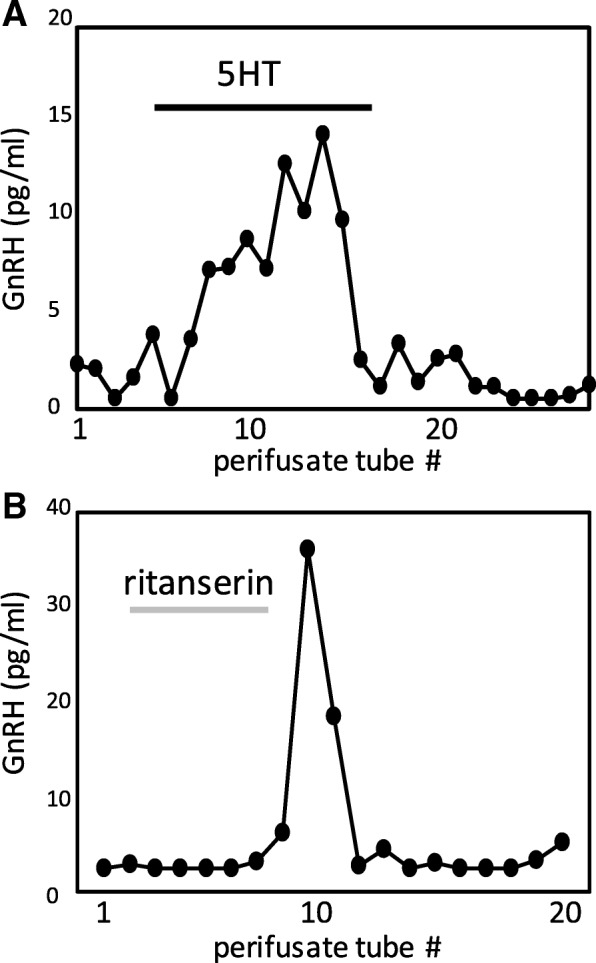
**a** Representative plots of GnRH secretion from perifused COS treated with 12.5 μM 5HT for duration indicated by black bar. **b** GnRH secretion from perifused COS treated with 6.0 μM 5HTR2A-specific inhibitor ritanserin for duration indicated by grey bar

Interestingly, exposure of perifused COS cells to 12.5 μM 5HT also rapidly and transiently increased GnRH secretion (Fig. 6a), while treatment with the specific 5HTR2A inhibitor ritanserin blunted GnRH secretion, followed by a rebound in secretion during inhibitor washout (Fig. 6b). These results suggest that 5HT may also act on the release of GnRH as a positive feedback loop within osteosarcoma tumors.

## Discussion

Canine and human osteosarcoma unfortunately often develops rapidly to terminal stages due to metastases to lungs and other sites [3]. Thus, elucidation of mechanisms used by these tumors to maintain proliferation rate and establish independence within their specific microenvironment could be helpful in establishing targeted therapies. In this study, we identified two peptides, GnRH and kisspeptin, typically found as key signals within the neuroendocrine reproductive axis, exerting effects on cultured canine osteosarcoma tumor cells in vitro, and further found that the normally-observed stimulatory regulation of kisspeptin on GnRH secretion is recapitulated within these cells. In addition to being expressed in established osteosarcoma lines, GnRH also appears to be expressed in primary tumor samples from canine patients. Secretion of GnRH from COS was detected using radioimmunoassay, demonstrating that these cells can secrete this decapeptide in measurable amounts, and that this basal release of GnRH could be stimulated by exposure to human kisspeptin. While this current study also reveals that a few canine osteosarcoma cell lines synthesize *kiss1*, the possibility cannot be excluded that circulating levels of kisspeptin from the liver or other distal or proximal sites may typically act on cognate Kiss1R in these tumors. *Kiss1* expression has been previously noted in human osteosarcoma cell lines and adjacent tissues [16] as well as in other cancer cell types [29–31]. Canine kisspeptin appears to be markedly different from the human and murine version of the peptide [26], and while we were able to detect expression in some tumor lines via RT-PCR, it is unclear if the peptide is synthesized, since a canine-specific ELISA is currently unavailable. As normal canine osteogenic progenitor cells appear to possess significantly lower levels of *Kiss1R* expression, it would seem that tumor cells may selectively up-regulate this receptor, and that Kiss1R signaling is less frequently observed in a normophysiologic context. The expression of *kiss1r* observed in normal canine osteogenic progenitor cells could be due to the transcription factor Runx2 activating *kiss1r*, which is consistent with OPCs expressing *runx2* as a function of osteoblastic lineage commitment [32]. Additionally, previous studies have observed variable expression levels of the *kiss1r* transcript in normal osteogenic progenitor cells and mesenchymal stem cells, resulting in varying amounts of Kiss1R protein synthesis, occasionally including no detectable protein [33]. In other cancer types, several studies demonstrate that kisspeptin may act as a tumor suppressor [34–36], while a handful of recent studies suggest that Kiss1R activation may promote breast tumor proliferation and metastasis [37–39].

Additionally, while we observed effects of GnRH in osteosarcoma cell lines in the form of changes in proliferation and gene expression, the pituitary-typical transcript of GnRHR was found to have a significantly low basal abundance in COS cells. This is consistent with the normophysiologic effect of downregulating *gnrhr* expression in response to continuous GnRHR stimulation by GnRH. Potential induction of *gnrhr* expression by kisspeptin and/or pulsatile GnRH treatment suggests that autocrine and paracrine signaling, mediated by endogenous production of these peptide hormones by tumor cells, is sufficient to maintain adequate transcript abundance for GnRHR protein production, which could subsequently sensitize COS cells to further GnRH. In support of this, GnRH effects on proliferation are reversed by application of the GnRHR antagonist Teverelix suggesting that the receptor is not desensitized in the presence of constitutive GnRH production and non-pulsatile GnRH secretion.

While some studies of human osteosarcoma and breast cancer suggest that GnRH may decrease metastasis [12, 40], our data, in contrast, suggest a model by which autocrine production of GnRH may allow canine bone cancer an additional way to maintain and progress. One hallmark of cancer is sustaining proliferative signaling [41, 42], which can be provided by autocrine or paracrine signals that override normal apoptotic mechanisms, tilting the balance toward unregulated cell proliferation. We have previously shown that 5HT can act at HTR2A, which are strikingly upregulated in osteosarcoma cells, to influence growth rate [8]. Our current model posits that minor amounts of circulating 5HT can induce production and/or release of GnRH from tumor cells, which can be potentiated by either autocrine or circulating kisspeptin. Increases in GnRH exposure could then allow for tumor proliferation, partially by increasing *htr2a* expression, thus leading to a positive feedback growth response upon subsequent exposure to enteric 5HT. Circulating and/or autocrine kisspeptin could alter tumor proliferation rate by stimulating autocrine GnRH release. Gpr54/Kiss1R expression was observed in normal osteoblast cells at levels significantly lower than what was found in tumor lines. While not fully confirmed, this suggests that Kiss-1 signaling in cancer cells may act both as a growth factor (via increases in the autocrine production of GnRH), and a tumor suppressor (independently of GnRH). Kisspeptin treatment in the presence of blockade of GnRH action by Teverelix resulted in a decrease in proliferation compared to that observed with kisspeptin alone, further suggesting that Kiss-1 may increase proliferation in a GnRH-dependent manner.

COS cells are primarily osteoblastic, yet express both *rank* and *rankl* (in addition to *opg*), potentially conferring the ability to signal in autocrine and paracrine manners to modulate proliferative rate. Thus, factors that may potentially modulate *rank*, *rankl*, *and opg* expression – such as GnRH, kisspeptin, 5HT, and estrogen – may further influence tumor progression, due to the anti-apoptotic and pro-proliferative effects of NF-κB activation. Our data indicate that GnRH exposure increases *rankl* expression, which may account for its observed effect to increase proliferation. This effect appears to be receptor-specific, as it was also negated by Teverelix, which suggests an unknown mechanism of *rankl* potentiation exists downstream of GnRHR activation. Despite the eventual activation of NF-κB via RANKL, the expected downregulation of *gnrh* expression by NF-κB may be negated due to the presence of autocrine, paracrine, and circulating 5HT and/or kisspeptin, which would continue to promote *gnrh* expression. Interestingly, estrogen treatment elicited no additional changes in *opg* expression under any peptide hormone treatment, denoting a significant loss of normal osteoblastic behavior. COS cells are known to express ERα, which indicates they should be able to respond to circulating estrogen and subsequently increase *opg* expression. Failure to do so after estrogen treatment denotes a second aspect of dysregulation in bone remodeling. By increasing the RANKL/OPG ratio, irrespective of the presence of estrogen, GnRH and kisspeptin may thus disrupt bone remodeling homeostasis to favor osteoclastic activity, which may further accelerate the growth and spread of osteosarcoma.

Due to limitations inherent in in vitro model studies, further exploration is required to more completely determine what effects locally-produced kisspeptin and GnRH are exerting within the tumor microenvironment, including investigating changes in downstream signaling, and what similarities and differences exist between our findings and what may be found in an in vivo context. While many mechanistic details remain to be addressed, our current studies raise the possibility of targeting this traditionally-associated neuroendocrine signaling pathway, as well as RANKL protein, in a therapeutic manner for the treatment of osteosarcoma in dogs and potentially humans as well.

## Conclusions

In this study, we found that canine osteosarcoma cell lines and primary tumor samples express neuroendocrine hormones and receptors typically associated with the reproductive axis, such as *gnrh, kiss1*, *gnrhr*, and *kiss1r*, at differential levels in comparison to normal osteogenic progenitor cells. These expression patterns were associated with a functional sensitivity to autocrine, paracrine, and/or circulating GnRH and kisspeptin, as revealed by kisspeptin-induced GnRH secretion from COS cells. Further, proliferation of osteosarcoma cells was significantly altered by reproductive hormone exposure, as treatment of COS cells with exogenous kisspeptin, GnRH, and 5HT exerted pro-proliferative effects, which may be potentiated by increases in *rankl* and *5htr2a* expression. Lastly, exogenous 5HT was also found to increase GnRH secretion, establishing an additional positive feedback loop that may drive constitutive growth and signaling. Pro-proliferative effects were reversed by treatment with GnRHR and 5HTR2A antagonists, suggesting that these neuroendocrine hormones should be included in a list of viable targets for the treatment of osteosarcoma.

## Additional files

Additional file 1: **Figure S1.** Comparison of nucleotide sequences of *kiss1* (A) and amino acid sequences of Kiss-1 (B) in the dog, human, and mouse. (PDF 462 kb)

Additional file 2: **Table S1.** Nucleotide sequences of specific PCR primers. (PDF 336 kb)

## Abbreviations

COS: HMPOS, POS, D17, C4, canine osteosarcoma cell lines
GnRH: gonadotropin-releasing hormone
*kiss1*: kisspeptin gene
*opg*: Osteoprotegerin
*rank*: Receptor Activator of Nuclear Factor κ-B
*rankl*: RANK-Ligand
