# Supplementary material for: Autocrine production of reproductive axis neuropeptides affects proliferation of canine osteosarcoma in vitro
Source: BMC Cancer. 2019 Feb 18;19:158. doi: 10.1186/s12885-019-5363-4 (PMC6379937; doi:10.1186/s12885-019-5363-4)

Supp Figure 1

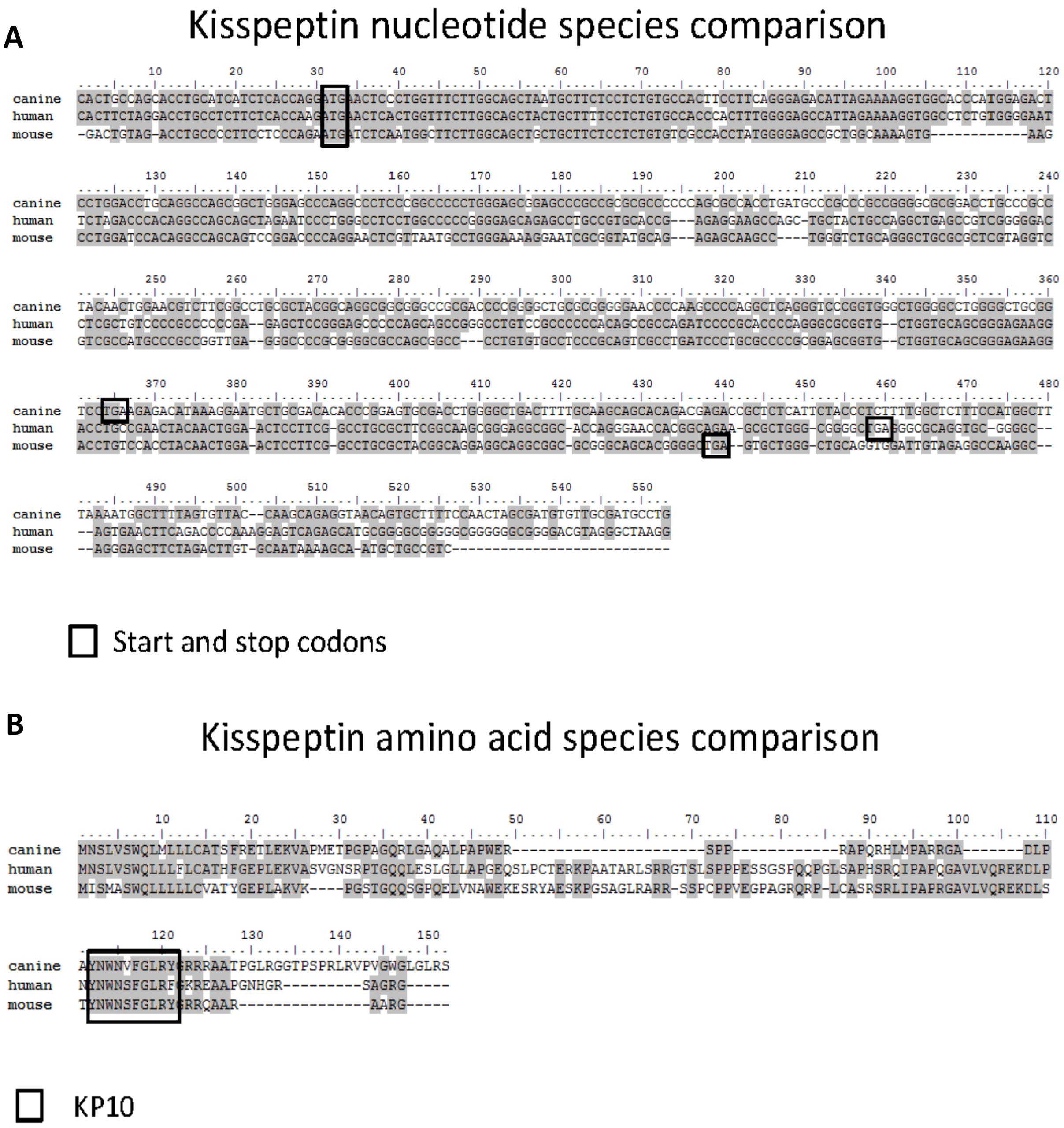

Supplement: Supplementary file 1 — Figure S1. Comparison of nucleotide sequences of kiss1 (A) and amino acid sequences of Kiss-1 (B) in the dog, human, and mouse. (PDF 462 kb) [file 12885_2019_5363_MOESM1_ESM.pdf]
