# Supplementary material for: Autocrine production of reproductive axis neuropeptides affects proliferation of canine osteosarcoma in vitro
Source: BMC Cancer. 2019 Feb 18;19:158. doi: 10.1186/s12885-019-5363-4 (PMC6379937; doi:10.1186/s12885-019-5363-4)
Supplement: Supplementary file 2 — Table S1. Nucleotide sequences of specific PCR primers. (PDF 336 kb) [file 12885_2019_5363_MOESM2_ESM.pdf]

Supplemental Table 1. Nucleotide sequences of specific PCR primers

Gene	Primer Sequence 5' – 3'	Product Size
cKiss1	F: GAAAAGGTGGCACCCATGGAGA<br>R: GCCGAAGACGTTCCAGTTGTAG	165 bp
cKiss1R	F: CGCCACCTGTGCCTGCTA<br>R: CCGCTCTGCCAGCAGCTG	106 bp
cGnrh	F: GGCCAACACTGGTCCTATGG<br>R: CCTCTTCAATCAGACTTTCCAGA	190 bp
cGnrhr	F: CCCCAGCCTTCATGATGGT<br>R: GGGATGATGAAGAGGCAGC	289 bp
cHtr1B	F: ACCTGCTCGTCTCCATCCT<br>R: TCCGACGACAGCCACAAGTC	103 bp
cHtr2A	F: CCCATTCTTCATCACGAACAT<br>R: GGAGAGGTAACCGATCCAGAC	106 bp
cRANK	F: CATTTCGGACGGTGCTGTAA<br>R: AAGTATTCATCCGGGCCACA	107 bp
cRANKL	F: CACTGACATCCCATCTGGTTC<br>R: CATTGCTGAAAGTCATGTTGGA	95 bp
cOPG	F: GGAGCTTGAGTTCTGCCTGAA<br>R: GGGTGCTTTAGATGACGTCTCA	139 bp
cβ-Actin	F: GGACCTGACCGACTACCTCAT<br>R: GGGCAGCTCATAGCTCTTCTC	181 bp
cYWHAZ	F: AGCCTGCTCTCTTGCAAAGAC<br>R: GGGTATCCGATGTCCACAATG	137 bp

[1]YWHAZ was used as a housekeeping gene to access quality of cDNA and to provide normalization standard for qPCR
